# Impaired of a non-DNA dependent methylation status decides the fat decision of bone marrow-derived C3H10T1/2 stem cell

**DOI:** 10.1186/2193-1801-2-590

**Published:** 2013-11-04

**Authors:** Faisal Ali, Yazan Ranneh, Amin Ismail, Bart Vaes

**Affiliations:** Department of Nutrition and Dietetics, Faculty of Medicine and Health Sciences, Nutrigenomics Programme, Universiti Putra Malaysia, 43400 UPM Serdang, Selangor, Malaysia; Metabolism and Genomics group, Division of Human Nutrition, Wageningen University, Bomenweg 2, 6703 HD Wageningen, The Netherlands

**Keywords:** Methylation mechanism, C3H10T1/2 stem cell, S-adenosylmethionine, S-adenosylhomo-cysteine, Osteablast, Adipocyte

## Abstract

A decrease in the lineage commitment of multipotent Mesenchymal stem cells (MSC) to the bone forming osteoblast lineage and an increase in the commitment to the fat forming adipocyte lineage is more common in bone marrow of elderly persons. A link between methylation status and MSC differentiation remains unclear. Therefore, we hypothesize that hypomethylation may decide the fate decisions of MSC. In the current study, murine bone marrow derived-C3H10T1/2 stem cell was used to examine the role of methylation mechanism on the differentiation potential of stem cells into osteoblasts or adipocytes. C3H10T1/2 cells were treated with Periodate oxidized adenosine (Adox), an inhibitor of S-adenosylhomocysteine-dependent hydrolase (SAHH), which in turn block the non-DNA methylation pathway. The effect of hypomethylation on C3H10T1/2 stem cell differentiation was determined by measuring the alkaline phosphates activity and the degree of mineralization as well as Oil-red O staining and lipid content. The ratio of S-adenosylmethionine (SAM) and S-adenosylhomocysteine (SAH) was determined as a metabolic indicator of cellular methylation potential. It was clearly observed that hypomethylation significantly (P < 0.05) reduces SAM: SAH ratio, alkaline phosphates activity, calcification and thereby, osteoblast differentiation. Conversely, adipocyte differentiation was stimulated by hypomethylation. Altogether, our data suggest that non-DNA hypomethylation changes the differentiation potential of C3H10T1/2 stem cells for less osteogenic and more adipogenic.

## Introduction

MSC are multipotent adult stem cells of mesodermal origin with the capacity to differentiate into cells of various multiple tissues, e.g., muscle, bone, cartilage, fat, and marrow stroma (Otto and Rao 2004). Multipotent MSC can be isolated from various adult tissues such as bone marrow (Conget and Minguell 1999), skeletal muscle (Asakura et al. 2001), adipose tissue (Zuk et al., 2002) umbilical cord blood (Romanov et al. 2003) and fetal lung (Anker et al. 2003). MSC can easily be harvested from bone marrow. When placed in culture, bone marrow-derived MSC retain their multipotential capacity and can differentiate into at least osteoblasts, chondrocytes and adipocytes (Pittenger et al., 1999). Methionine is converted to S-adenosylmethionine (SAM) by the enzyme methionine adenosyltransferase (MAT) during methylation pathway. SAM is the major biological methyl group donor to a large variety of acceptor substrates, including DNA, RNA, phospholipids, and proteins. S-adenosylhomocysteine (SAH) is formed, through donation of a methyl group, which is subsequently hydrolyzed to homocysteine by the enzyme SAH hydrolase (SAHH) (Hoffman et al., 1980; Chiang et al., 1996).

The ratio SAM/SAH is an important metabolic marker for cellular methylation status and also known methylation potential (MP) (Marina et al., 2004). Previous studies in vitro have been reported that inhibition of SAHH enzyme results in decreased SAM levels as a result of decreased methyltransferase activity leading to reduced SAM/SAH ratio in presence of significant increase in SAH levels (Marina et al., 2005; Rita et al., 2003). Furthermore, an altered SAM/SAH ratio by inhibition of SAH hydrolase results in inhibition of DNA, RNA, and protein and lipid methylation (Caudill et al. 2001). Thus, considering the crucial role of methylation in various cellular processes, it is known that any alteration in the availability of SAM may have profound impacts on cellular growth, differentiation, and function (Carmel and Jacobsen 2001). Previous studies as demonstrated in vitro and in vivo have provided evidence that chronic elevation in plasma Hcy associated with vitamins deficiencies have an indirect and negative effect on cellular SAH levels, which should result in feedback inhibition of SAM-dependent methyl-transferase reactions and reduced methylation capacity. This has to lead to the following hypothesis that homocysteine is a metabolic marker for a reduced methylation activity (Afman et al., 2005; Chen et al., 2001; Ping et al. 2000). The proper differentiation potential of Mesenchymal stem cell (MSC) into osteoblasts or adipocytes determines the balance between osteoblast and adipocyte number and activity, which is required to maintain bone and fat homeostasis. Therefore, disturbance of this equilibrium may contribute to the pathology of metabolic diseases such as ageing and osteoporosis (Elena et al., 2004; Toru et al., 2004). Research in recent years has been demonstrated that treatment of embryonic stem cells with retinoic acid induces a MSC-like intermediate cell that can subsequently be differentiated into osteoblasts, chondrocytes or adipocytes, but the underlying mechanisms remain to be solved. Moreover, the exact nature of progenitor cells that are responsible for the lineage commitment of mesenchymal stem cells is less well understood than in the case of hematopoietic stem cells due to a lack of specific markers to characterize intermediate stages. MSC and primary cultures need to be isolated freshly, thereby introducing possible variations between experiments as a result of cell isolations from different donors (Dani et al., 1997; Kawaguchi et al. 2005).

Clonal cell lines have the advantage that they provide homogenous populations that can often be maintained in culture for many passages. In order to study mechanisms of lineage specification, it is necessary to use cells with the flexibility to differentiate into multiple mesenchymal lineages. Examples of cell lines with this property are the adult mouse bone marrow-derived fibroblastic cell line C3H10T1/2 (10 T1/2) which has been derived from early mouse embryos. C3H10T1/2 stem cells can be stimulated by various external factors to differentiate into the three cell types osteoblasts, chondroblasts, and adipocytes (Otsuka et al. 1999; Ding et al. 2003).

The mechanisms that determine whether MSC differentiate into osteoblasts or adipocytes are still poorly understood. Recently, (Milhem et al., 2004) reported that DNA methylation plays an important role in the regulation of hematopoietic stem cell switching, but the link between methylation pathway and MSC differentiation remain ambiguous. Therefore, in this study it is hypothesized that the alterations in the commitment and differentiation potential of MSC during ageing may be explained by modification of non- DNA methylation pathway. Finally, to assess the role of demethylation potential in modifying stem cells fate decisions, SAH hydrolase in C3H10T1/2 stem cells was inhibited with peroidate oxidized adenosine (Adox) *in vitro*. The capacity of this agent to modulate osteoblast differentiation was analyzed under non-osteogenic control conditions and during growth factor-induced differentiation.

## Materials and methods

### Materials

Culture media (DMEM & αMEM), fetal bovine serum (FBS), L-glutamine, penicillin/streptomycin, and phosphate buffered saline (PBS) were obtained from (Lonza). Insulin from Novo Nordisk was used. The mouse embryo C3H10T1/2 fibroblasts cells were purchased from American Type Culture Collection (ATCC). The remaining tissue culture materials, 3-(4,5-dimethylthiazol-2-yl)-2,5-diphenyl tetrazolium bromide (MTT), dimethyl sulfoxide (DMSO) and periodate-oxidized adenosine (adenosine-2, 3-dialdehyde) (Adox) were obtained from Sigma Chemical Company Netherlands.

### Cell culture

In this study, C3H101/2 stem cells (10 T1/2)-derived from C3H mouse embryos were used. C3H101/2 stem cells are functionally similar to MSCs and have the potential to differentiate into multiple cell types, for example, osteoblasts, adipocytes or chondrocytes dependent on the stimulation agents that have been used. The cell culture took place in a humidified atmosphere containing 5% CO_2_ at 37°C. After reaching a confluency of 70-80%, all cells were rinsed with PBS (Lonza), treated with trypsin-EDTA (0.25 mg/dl) (Cambrex) until cells detached, and subsequently passed at lower density to new flasks in culture medium. Control medium consists of Dulbecco’s modified Eagle medium (DMEM) supplemented with 10% FBS, 2 mM L-glutamine, and 1% penicillin (100 U/ml) / streptomycin (100 μg/ml). The cells were cultured in presence or absence of series concentrations of Adox. Osteoblast differentiation (OB) medium consists of control medium supplemented with 12, 5 mM β-glycerophosphate, 50 μg/ml ascorbic acid and 1 mM dexamethasone. Adipocyte differentiation (AD) medium consists of control medium supplemented with 1 mM dexamethasone, 5 μg/ mL insulin, 111 μg/ml 3-isobutyl-methyl xanthine (IBMX) and 1 μM rosiglitazone was added as well. All cell lines in culture medium were seeded in DMEM/10% FBS at a density of 1.0 x 10^4^ cells/cm^2^. Differentiation was initiated two days later (t0) by removing the culture medium and replacing it with either control medium, osteoblast differentiation medium or adipocyte differentiation medium as supplied by the manufacturer. From t0, cells were exposed to series concentrations of Adox.

### Cell viability by MTT assay

Cell death or cytotoxicity is classically evaluated by the quantification of plasma membrane damage. The cytotoxicity of Adox on seeded C3H10T1/2 stem cells was colorimetrically evaluated by measuring the formation of formazin salts because of the reduction of 3-(4,5-dimethylthiazol-2-yl)-2,5-diphenyl tetrazolium bromide (MTT) reagents. The increase in the amount of formazan produced in culture supernatant directly correlates to the increase in the number of lysed cells. The formazan dye is water-soluble and can be detected by spectrophotometer at 570 nm. The MTT-cytotoxicity assay is sensitive, convenient, and precise, and is applicable to a variety of cytotoxicity studies.

The cells were cultured for 24 hrs. Then, Adox in different concentrations (10-100 μM) were added. After 24 and 72 hours incubation, 20 μl of 5 mg/ml MTT reagents were added to each well. Then, the wells were incubated for 4 h at 37°C and the formazen crystals were dissolved by adding 200 μl of dimethyl sulfoxide (DMSO) at 37°C for 30 min. The optical density of the wells was read at 570 nm on a microplate reader (Molecular Devices Inc., Sunnyvale, CA, USA). The rate of cell death was specified relative to control group.

### Determination of SAM and SAH levels

Measurement of SAM and SAH concentration in culture medium was done according to the method of (Dani et al. 1997). The SAM and SAH was briefly extracted from cultured cell, and then 80 μl of o.1 M sodium acetate buffer, pH 6.0, was added to a soft pellet containing a known number of cells. After the cells were lysed with the sodium acetate, 20 μl of 40% TCA were added. The solution was vortex-mixed and allowed to stand on ice for 30 min. The solution was centrifuged at 25,000 g for 10 min at 5°C, and 100 μl of the supernatant were transferred to another 0.5 ml eppendorf tube. The solution was extracted twice with an equal volume of diethyl ether, and was filtered through a 0.2- μm filter. Then, the solution was injected into the high-performance liquid chromatography (HPLC) system. Detection of SAM and SAH was performed using a CoulArray electrochemical detector (ESA, Chelmsford, MA). Peak area analysis was carried out by HPLC based on calibration curves generated for each compound.

### Alkaline phosphate assay

To measure the progress of osteoblast differentiation in C3H10T1/2 cultured cells, the activity of the protein Alkaline Phosphatase (ALP) assay was measured. Briefly, cells were cultured in 96-wells plates and medium conditions were applied in triplicate. A spectrophotometic analysis of ALP protein activity was performed at various time points. The cells were rinsed with cold PBS, and fixed with 4% paraformaldehyde for 10 minutes at 4°C. Subsequently, a dilution of P-nitrophenyl phosphate (pNPP) (Sigma) was used based on the type of cell line. Substrate for ALP was added and the plate was incubated at 37°C for 30 minutes. The color reaction, taking place through the conversion of pNPP to p-nitrophenol by ALP, was stopped by addition of NaOH and the extinction was measured at 405 nm with a MultiSkan Ascent spectrophotometer (Thermo Labsystems) and version 2.4.2 MultiSkan Ascent software.

### Neutral red assay

To correct for cell numbers during the ALP assay, a Neutral Red (NR) assay was carried out. This assay is always performed in combination with ALP assay that so-called ALP/NR assay to correct the cell numbers by dividing the result of the ALP assay by the result of the NR assay. Based on manufacture’s protocol, cells were seeded in 96-wells plates and medium conditions were applied in triplicate. Neutral Red (Sigma) (1 mg/ml) was diluted in PBS, then added to each well of a 96-wells plate and the plates were incubated at 37°C for one hour. Subsequently the cells were rinsed with PBS and the remaining absorbed NR was released by adding an elution solution. Extinction measurements were performed at 550 nm on a MultiSkan spectrophotometer.

### Alkaline phosphatase / Oil-red-O staining

To visualize the amount of osteoblasts and adipocytes, alkaline phosphatase / Oil-red-O staining (ALP/ORO) was used. Briefly, Oil-red-O staining was performed on distinct time points. The cells were fixed for 30–60 seconds with citrate-buffered acetone (kit from Sigma-Aldrich) and then washed twice with MilliQ water (MQ). ALP staining was performed by adding a dye mixture of diazonium salt solution and Naphtol AS-MX phosphate alkaline solution (Sigma-Aldrich). The cells were incubated in the dark for 30 minutes to 3 hours at 37°C until the blue staining was visible. After which they were rinsed with MQ water. For subsequent lipid droplets staining, an Oil-Red-O (Sigma) working solution was added and the plate was incubated for 15 minutes at 37°C. The cells were rinsed once again with MQ and photographs were taken under the microscope (Olympus CKX41) using an Olympus C-5050 Zoom camera and Olympus DP-soft version 3.2 software.

### Quantification of mineralization

This assay is performed to measure the amount of calcium deposited, which is formed during the later stage of osteoblast differentiation as an indicator for the level of calcification or mineralization. Cells were cultured in 24-wells plates and medium conditions were applied. Calcium deposition was measured with a calcium release assay when the whole time-series was isolated. The cells were incubated with HCL (0.5 M) and shaken overnight. A color reaction was performed by adding 25 μl of the collected and centrifuged HCL samples to a mix of o-cresolphthalein complexone, 8-quinolinol and 2- amino-2-methyl-1, 3-propanediol (Sigma) in cuvettes. A blank (25 μl HCL) and a standard series of calcium solution, were included. Extinction was determined at 575 nm on a UV mini 1240 spectrophotometer (Shimadzu).

### Quantification of lipid

A triglyceride assay was performed to measure the degree of adipocyte differentiation and lipid accumulation. Since, adipocytes are a highly specialized cell type included in the store and accumulate lipid as triglyceride in response to adipogenic factors such as insulin, dexamethasone, rosiglitazone and IBMX. Briefly, cells were seeded in 24-wells plates and medium conditions were applied in duplicate followed by rinsing with PBS, then incubated with a Tris–HCl EDTA solution at -20 until all samples were collected.

Plates were brought to room temperature to determine triglyceride content according to the protocol. A standard series was included by adding triglyceride standard (kit from iNstrucchemie, ref # 10720P) to wells without cells. 40 μl Tertiary butanol and 10 μl methanol were added to the wells to extract the triglycerides from the cells, and shaking for 10 minutes. A color reaction was performed by adding the reagent triglycerides Liquicolour Mono (kit from iNstruchemie, ref #10720P). After shaking for 20 minutes, samples were pipetted into a 96-wells plate and extinction was measured at 492 nm with a MultiSkan spectrophotometer.

### Statistical analysis

Data are expressed as the mean ± standard deviation. One way-ANOVA (SPSS version 20.0) and LSD post hoc test were used to determine the mean differences between groups. Values are considered significantly different at the level of < 0.05.

## Results

### Cell cytotoxicity

**Figure 1:**
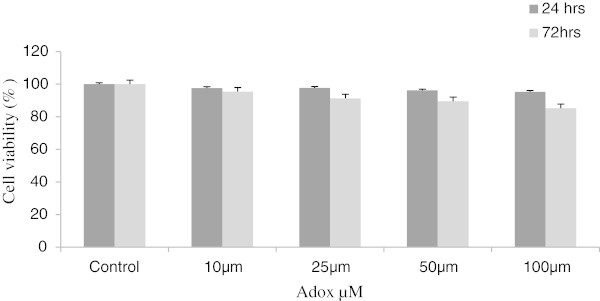
**Effect of Adox on cell cytotoxicity in C3H10T1/2 stem cells.** Cells were treated with different concentrations of Adox for 24 and 72 hrs. Cells in control were cultured in OB medium treated with (DMSO) as a solvent control (the final concentration of DMSO was 0.2%). The other cells were cultured in OB medium containing different concentrations of Adox (10-100 μM). Cell cytotoxicity was determined by MTT assay and expressed as a percentage of viable cells in the total number of cells counted. Values are means ± SD (n = 3) for each treatment.

Incubation with 10–100 μM Adox for 24 and 72 hrs, did not show any cell cytotoxicity when compared with (DMSO) as a solvent control (the final concentration of DMSO was 0.2%) as shown in Figure 1.

### SAH and SAM levels

To investigate whether intracellular SAH and SAM are involved in the non-DNA hypomethylation, we measured intracellular SAH and SAM during incubation with various concentrations of Adox (10-50 μM). As shown in (Table 1), incubation with DMEM and OB medium (as a negative control) did not change intracellular SAH and SAM levels in C3H10T1/2 stem cells. However, incubation of cells with 10–50 μM Adox significantly enhanced increased intracellular SAH levels (3.99 ± 0.19 nmol/L) compared with (1.6 ± 0.48 nmol/L) in controls. SAM/SAH ratio was also determined in cells incubated for 48 h with Adox (Table 1). Incubation of C3H10T1/2 stem cells with 50 μM Adox decreased the SAM/SAH ratio in culture medium (18.2 ± 0.26 to 8.5 ± 0.18, p < 0.05).

**Table 1:** **Effects various concentrations of Adox on content of SAM, SAH and SAM: SAH ratios for 48 h in C3H10T1/2 stem cells** ^**1**^

Treatment	SAH^2^ (nmol/L)	SAM (nmol/L)	SAM: SAH ratio
Control	1.6 ± 0.48^a^	28 ± 0.41^b^	17.5 ± 0.28^c^
DSMO + OB	1.7 ± 0.38^a^	31 ± 0.32^b^	18.2 ± 0.26^c^
Adox + OB			
10 μM	1.9 ± 0.12^a^	35 ± 0.25^c^	18.4 ± 0.16b^a^
25 μM	2.66 ± 0.14^b^	33 ± 0.29^a,b^	12.4 ± 0.14^c^
50 μM	3.99 ± 0.19^c^	34 ± 0.23^b^	8.5 ± 0.18^b,c^

^1^Cells in the control condition were only exposed to control medium without any osteogenic supplements, the other cells were cultured in OB medium treated with (DMSO) as a solvent control (the final concentration of DMSO was 0.2%). Cells in OB (osteogenic medium) exposed to OB medium without Adox treatment. The other cells were cultured in OB medium containing different concentrations of Adox (5-50 μM). ^2^Values are means ± SD of triplicate assays. Data in the column with different superscript letters are significantly different (P < 0.05).

### Osteoblast differentiation

Continuous treatment the confluent cells with differentiation inducers resulted in terminal osteoblast differentiation. Adox was used as a potent methylation inhibitor because it has been shown to inhibit methylation pathway in previous studies (Afman et al., 2003; & Afman et al. 2005).

### ALP staining

**Figure 2:**
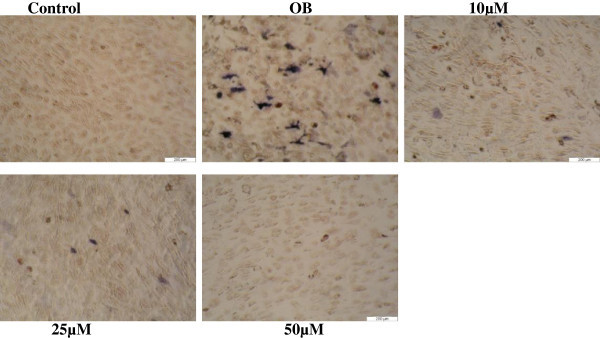
**Photographs of C3H10T1/2 stem cells treated with Adox on day 11.** All cells were stained with alkaline-dye, according to the protocol described in material and methods. All cells in the control were only exposed to control medium without any osteogenic supplements. Cells in OB (osteogenic medium) exposed to OB medium without Adox treatment. The other cells were cultured in OB medium containing different concentrations of Adox (10-50 μM).

Cells were stained with alkaline-dye to visualize the amount of differentiated osteoblasts from MSCs. Cells were cultured in control medium without any supplements and OB medium supplemented with (ascorbic acid, beta-glycerophosphae, vitamin D and dexamethasone) in the presence of series concentrations of Adox (5 - 50 μμ) and then stained for ALP-positive cells using ALP staining on day 11, the resulting pictures are presented in Figure 2.

The blue staining indicates osteoblast differentiation. The pictures show that in OB medium large numbers of the human stem cells were differentiated into osteoblasts that express high amount of ALP compared with those in control medium due to the potent effect of osteogenic supplements that were used for osteoblast differentiation. A marked decrease in the number of ALP-positive cells treated with series concentrations of Adox compared with the number of positive cells in untreated osteogenic medium (OB) can be observed. This result indicates that osteogenesis is decreased due to the inhibition of methylation pathway in C3H10T1/2 stem cells.

### Alkaline phosphatase activity

**Figure 3:**
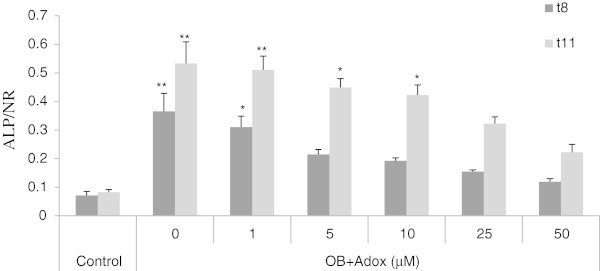
**ALP/NR assays of C3H10T1/2 stem cells treated with Adox.** The assays were performed on t8 and t11 respectively, according to the protocol in materials and methods. Cells in the control condition were only exposed to control medium without any osteogenic supplements. Cells in OB (osteogenic medium) exposed to OB medium without Adox treatment. The other cells were cultured in OB medium containing different concentrations of Adox (5-50 μM). Results are expressed as means ± SD (n = 3). *P < 0.05, **P < 0.01 in comparison with control group values.

To quantify the result of osteoblast differentiation in cells, ALP assay was done. Cells were treated with different concentrations of Adox (5–50 μM). The influence of Adox on the synthesis of ALP by osteoblasts was determined by measuring ALP activity, corrected for cell number as determined by neutral red adsorption. ALP activity was measured on day 8 and day 11 respectively, and the resulting graph is shown in Figure 3. As observed by spectrophotometric measurement, ALP activity is strongly induced in osteogenic medium compared with control medium. A marked decrease in the ALP activity compared with the ALP activity in osteogenic medium can be observed in the cells that have been treated with series concentrations of Adox.

### Mineralization

**Figure 4:**
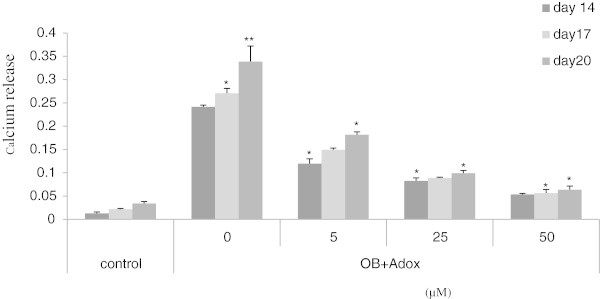
**Calcium release assay of C3H10T1/2 treated with Adox.** The assays were performed on day 14, day 17 and day 20 respectively, according to the protocol in materials and methods. Cells in the control condition were only exposed to control medium without any osteogenic supplements, the other cells were cultured in OB medium. Cells in OB (osteogenic medium) exposed to OB medium without Adox treatment. The other cells were cultured in OB medium containing different concentrations of Adox (5-50 μM). Results are expressed as means ± SD (n = 3). *P < 0.05, **P < 0.01 in comparison with control group values.

To investigate the effect of inhibition of the methylation pathway by Adox on matrix calcification or mineralization, a calcium release assay was performed in cells. Cells were grown in control and osteogenic medium for 20 days and the calcium release assay was measured on t14, t17, and t20. From the results shown in Figure 4, it can be stated clearly that osteogenic agents have stimulatory effect on calcium release in osteogenic medium compared with control medium. While, Adox in treated cultures has strong an inhibitory effect on calcium release at every time points compared with osteogenic medium. A dramatic repression is achieved on exposure to 50 μM of Adox, which disables the cells to calcify at all.

### Adipocyte differentiation

**Figure 5:**
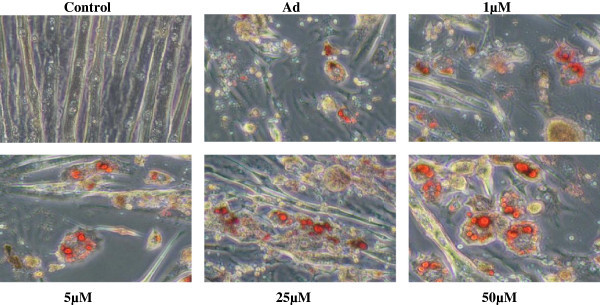
**Photographs of C3H10T1/2 stem cells treated with Adox on day 14.** All cells were stained with Oil-red-O, according the protocol described in material and methods. All cells in the control were only exposed to control medium. Cells in Ad (Adipogenic medium) exposed to Ad medium without Adox treatment. The other cells were cultured in Ad medium containing different concentrations of Adox (5-50 μM).

The confluent cells were cultured in control medium lacking adipogenic agents and in adipogenic medium containing differentiation inducers dexamethasone, insulin, rosiglitazone and isobutylmethylxanthine. Cells treated with different concentrations of Adox (5-50 μμ) and then stained for lipid droplets using an ORO staining on day 9, the resulting pictures are presented in Figure 5.

### ORO staining

The red spots indicate accumulation of lipid droplets, serving as an indication of adipocyte differentiation. The pictures clearly show that in adipogenic medium, C3H12T1/2 cells were differentiated into adipocytes that produce lipid droplets compared with those in control medium due to the potent effect of adipogenic supplements that were used for adipogenic differentiation. However, higher numbers of C3H10T1/2 stem cells were differentiated into adipocytes in the cells that have been treated with series concentration of Adox compared with Ad medium lacking Adox treatment. Interestingly, the treated cells with a higher concentration of Adox have been filled with an extensive accumulation of lipid droplets compared with adipocytes in Ad medium.

### Lipid quantification

**Figure 6:**
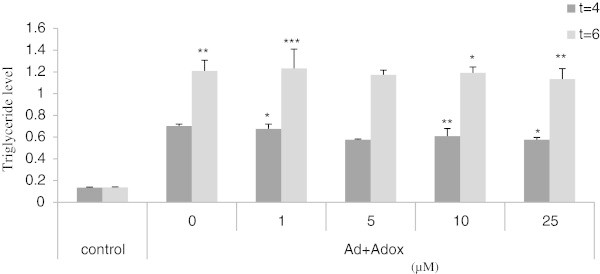
**Triglyceride of C3H10T1/2 stem cells treated with Adox on day 4 and day 6 respectively.** All measurements were done as duplicate and averaged, according to the protocol described in material and methods. All cells in the control were only exposed to control medium. Cells in Ad (Adipogenic medium) exposed to Ad medium without Adox treatment. The other cells were cultured in Ad medium containing different concentrations of Adox (5-50 μM). *P < 0.05, **P < 0.01, ***P < 0.01 in comparison with control group values.

To quantify the result of adipocyte differentiation of C3H10T1/2 stem cells, a triglyceride assay was carried out. The result is shown in Figure 6, and a great similarity with ORO staining can be observed especially at t4 and t6. At every time point a positive effect of adipogenic agents on adipocyte differentiation was seen compared with control medium. Whereas, Adox treatment clearly results in induction of the amount of triglycerides in a dose dependent way in treated cells compared with those in adipogenic medium.

### Osteoblast and adipocyte differentiation

**Figure 7:**
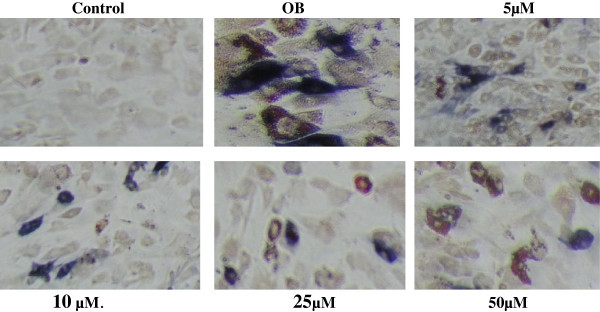
**Photographs of C3H10T1/2 stem cells treated with Adox on day 11.** All cells were stained with alkaline-dye mixture and Oil-red-O, according to the protocol described in material and methods. All cells in the control were only exposed to control medium without any osteogenic medium. Cells in OB (osteogenic medium) exposed to OB medium without Adox treatment. The other cells were cultured in OB medium containing different concentrations of Adox (5-50 μM). The other cells were cultured in OB medium containing different concentrations of Adox.

We next studied the effect of Adox on osteogenesis and adipogenesis simultaneously to assess the role of methylation pathway on the osteoblast and adipocyte differentiation together. The C3H10T1/2 stem cells were used and stained with alkaline-dye mixture and ORO staining. The result is shown in Figure 7 respectively. The blue staining indicates osteoblast differentiation, whilst the red spots indicate accumulation of lipid droplets inside adipocyte differentiation. Although the medium was meant to trigger osteogenesis, the adipocyte formation was grown in OB medium.

## Discussion

In the present study we have investigated the role of hypomethylation in commitment and cellular differentiation of multipotent C3H10T1/2 stem cells as characterized by SAM/SAH ratio, ALP/NR, calcium release, triglyceride assays and ALP/ORO staining. Our results clearly showed that that murine C3H10T1/2 cells have many features of mesenchymal stem cells. Also, it was demonstrated that non-DNA hypomethylation reduces stem cells differentiation into mature osteoblasts and therefore, inhibits osteogenesis in stem cells; this indicates that hypomethylation acts as a negative regulator of osteoblast differentiation. Moreover, this study demonstrated that the inhibitory effect of hypomethylation is not restricted on the early stage of stem cells but also this effect persists throughout the later stage of osteoblast differentiation as has been shown in the level of calcification that already committed to osteoblast lineage Figure 4. The results obtained from calcium release in C3H10T1/2 stem cells were very interesting and showed that hypomethylation also inhibits mineralization of matrix proteins that is the major function of osteoblast. On the other hand, demethylation obviously enhances stem cells differentiation into mature adipocytes and therefore, induces adipogenesis in C3H10T1/2 cells. This result is in line with other study performed in embryonic stem cells reported that DNA hypomethylation associated with lipid deposition in the proximal portion of the aorta of mice in vivo (Chen et al., 2001). But, C3H10T1/2 cells don not show any stimulatory or inhibitory effect under the influence of demethylation in the presence of rosiglitazone. However, another study showed that blocking DNA methylation by using another methylation inhibitor called Aza in C3H10T1/2 stem cells without roziglitazone, resulted in stimulation of adipogenesis after culturing for 28 days (Taylor and Jones, 1979). One of the explanations is that adipocte differentiation was very strong due to the strong effect of rosiglitazone a potent inducer of adipocyte differentiation. That means if Adox would stimulate adipocyte formation it cannot be detected. Furthermore, the influence of demethylation on osteogenesis and adipogenesis were studied simultaneously Figure 7. It was observed that demethylation is associated with a reciprocal decrease of osteogenesis and an increase of adipogenesis. These findings clearly show the role of methylation pathway as a key regulator of stem cell differentiation switch into less osteogenic and more adipogenic cells. Since, demethylated stem cells failed to differentiate into osteoblasts but differentiated into adipocytes in OB medium of stem cells, which has been used to trigger osteogenesis. The high degree of adipogenesis in Figure 7 was unexpected. The inverse relation between the number of osteoblasts and the number of adipocytes under the effect of demethylation reveals the importance of the methylation pathway in MSC for proper commitment and differentiation. In addition, the role of methylation pathway as a key regulator for stem cell differentiation is also observed in hematopoietic stem cell (HSC) (Milhem et al., 2004). after being treated with Aza a potent DNA methylation inhibitor and TSA as histone deacetylation inhibitor resulting in a significant alteration in the fate of HSC (Boer et al. 2006). The ratio of SAM to SAH (the substrate or product) of most cellular methyltransferase reactions is generally considered to be predictive of cellular methylation mehanism. Several studies have confirmed the decrease in SAM as the major effector of the reduced ratio and of methylation mechanism (Lathrop Stern et al. 2000; Loehrer et al., 1996; Simile et al., 1994).

The primary importance of SAH as a mediator of methylation status was recently reported in a human study in which it was found that increased levels of plasma homocysteine and intracellular SAH, without concurrent changes in SAM, were associated with lymphocyte DNA hypomethylation (Yi et al., 2000). In the present study, Adox methylation inhibitor was used to modulate SAM and SAH concentrations in C3H10T1/2 stem cell model to determine which intracellular metabolite has a greater effect on non- DNA methylation and determine whether stem cells differentiate into osteoblasts or adipocytes. The relationship among SAM, SAH and non-DNA hypomethylation observed in the culture medium confirmed the importance of SAH as the primary determinant of reduced methylation capacity (see Table 1).

These findings support the concepts that methylation modification may play an important role in the differentiation switching of stem cells. In this study it must be mentioned that the present results of osteoblast differentiation by our model systems are not in agreement with previous studies that showed that DNA demethylation by Aza agent in C3H10T1/2 cells and MG63 cells is associated with elevation of ALP expression and mineralization and therefore, osteoblast differentiation (Boer et al., 2006; Rachel et al., 1998). A possible explanation for the lowered osteoblast differentiation in our study after treated the cells with Adox could be resulted from demethylation of protein, lipid or RNA but DNA methylation might not be involved in the negative effect of demethylation. Since, that DNA methylation is not influenced by short-term alterations in methylation potential as has been observed previously by (Marina et al. 2004), who reported that a reduced methylation potential in HepG2 cells by Adox does not change the global DNA methylation while, mRNA methylation is reduced within 24 h. Moreover, others showed a clear reduction in protein methylation after Adox treatment (Delong et al., 2002; Li et al., 1998). However, in our study we do not examine global DNA methylation but based on the literatures we propose that DNA methylation could be not changed via inhibition of methyl acceptors that are involved in the process of cellular differentiation of MSC. Since, Adox was used to block methylation of lipid and protein but not DNA or RNA, indirectly by inhibition of SAH hydrolase enzyme in methylation cycle, therefore, it is not known exactly which methyl acceptor is inhibited and caused reduction for osteoblast differentiation. But, Aza in the previous studies has been directly incorporated into DNA molecule and thereby prevent methylation of DNA in the replicated stem cells.

Although these data support the possibility that alteration in the balance between osteoblast and adipocyte differentiation during osteoporosis and ageing might be mediated by hypomethylation. Further studies are needed to clarify the influence of an altered of methylation pathway on DNA, mRNA or protein methylation separately to elucidate whether the effect is due to a changed methylation of DNA, mRNA or due to the modification of protein methylation. Furthermore, determination of DNA hypomethylation levels and epigenetic regulation of stem cells is highly recommended as robust parameter for cellular methylation status.

## Conclusion

In summary, the results of this study demonstrate that hypomethylation changes the differentiation potential of C3H10T1/2 stem cells for more adipogenic and less osteogenic. Our results also show that epigenetic regulation of mesenchymal stem cell could open new strategy for therapeutic intervention of osteopenic disorders. However, further studies are still needed to investigate the influence of global DNA hypomthylation on the differentiation potential of mesenchymal stem cell markers in *vitro* and *vivo*.
